# Fungal, Bacterial, and Archaeal Diversity in the Digestive Tract of Several Beetle Larvae (Coleoptera)

**DOI:** 10.1155/2018/6765438

**Published:** 2018-04-16

**Authors:** Elvira E. Ziganshina, Waleed S. Mohammed, Elena I. Shagimardanova, Petr Y. Vankov, Natalia E. Gogoleva, Ayrat M. Ziganshin

**Affiliations:** ^1^Department of Microbiology, Institute of Fundamental Medicine and Biology, Kazan (Volga Region) Federal University, Kazan 420008, Russia; ^2^Department of Biotechnology, Faculty of Agriculture, Al-Azhar University, Cairo 11651, Egypt; ^3^Laboratory of Extreme Biology, Institute of Fundamental Medicine and Biology, Kazan (Volga Region) Federal University, Kazan 420021, Russia; ^4^Kazan Institute of Biochemistry and Biophysics, Kazan Science Centre, Russian Academy of Sciences, Kazan 420111, Russia

## Abstract

Interpretation of how partnerships between fungi, bacteria, archaea, and insects are maintained through the life of the hosts is a big challenge within the framework of symbiosis research. The main goal of this work was to characterize the gut microbiota in larvae of several Coleoptera species using sequencing of the bacterial and archaeal 16S rRNA genes and fungal internal transcribed spacer (ITS) region. Thus, larvae with various food preferences, including* Amphimallon solstitiale*,* Oryctes nasicornis*,* Cucujus cinnaberinus*,* Schizotus pectinicornis*,* Rhagium mordax*, and* Rhagium inquisitor*, were thoroughly investigated in this work. We revealed an association of these beetle species mainly with four bacterial phyla, Proteobacteria, Firmicutes, Actinobacteria, and Bacteroidetes, as well as with three fungal phyla, Ascomycota, Zygomycota, and Basidiomycota, but microbial communities varied depending on the beetle host, individual organism, and surrounding environment. Moreover, archaea within the phyla Euryarchaeota and Crenarchaeota in the hindgut content of* O. nasicornis* and* A. solstitiale* were additionally detected. The identified microbial communities suggest their potential role in the exploitation of various resources, providing nutritional needs for the host organism. These microorganisms can also represent a valuable source of novel metabolic capacities for their application in different biotechnologies.

## 1. Introduction

The order Coleoptera is one of the most abundant branches of insects. Representatives of this order have occupied a diverse range of ecological niches during their evolution. They consist of representatives of diverse groups, including Passalidae [1], Scolytinae [2], Cerambycidae [3], and Buprestidae [3, 4], which belong to wood-feeding beetles, Scarabaeidae which are mainly herbivorous or saprophagous [5], Silphidae which are known for their behavior to use vertebrate carcasses for nutrition [6], and many other taxa.

Beetles are found very interesting in their biology and especially in their interactions with different microbes. Although the anatomy and physiology of many beetles have been well investigated, their ecto- and endomicrobiome are overwhelmingly poorly understood. As various insects, beetles are closely associated with the microbial world, and their external cuticle and their digestive system are the most accessible habitats for microbial colonists. Such attributes of intestines as access to nutrients as well as protection against stress attacks of the external environment are considered attractable and favorable for microbial colonization. Furthermore, microorganisms are involved in the food digestion processes and can also be pathogens or antagonists of pathogens. Symbiotic microorganisms can additionally provide a source of essential amino acids, vitamins, and nitrogen. Different communities of bacteria and fungi are important partners of beetles, which ultimately affect the metabolism of the host [2, 3, 5–7]. Moreover, several archaeal representatives can also be distinguished within the beetles' gut microbiome [8, 9]; however, literature data describing the diversity of archaeal consortia are very limited.

High throughput sequencing approaches allow a better resolution of the existing diversity in different samples in comparison with culture-dependent techniques or gene cloning and sequencing methods [10–14]. Application of culture-dependent methods may lead to inaccuracy in the estimation of microbial diversity due to several factors, including specific characteristics of the growth of microorganisms. New advanced techniques make it possible to obtain a more detailed picture and allow researchers to focus on the microbiome of different species of invertebrates, including gut-associated microorganisms in larvae and adults of various beetles [1, 6].

Beetles belonging to the family Scarabaeidae include saprophagous beetles that thrive on rotting manure, humus, or decaying matter, phytophagous beetles that feed on the seeds, roots, and foliage of plants, as well as xylophagous beetles [8, 15]. In general, the intestinal tracts of various soil insects inhabit diverse microbial communities which can participate in various processes. For example, the most common bacterial class in the guts of adult insect and larval midgut of* Melolontha hippocastani* was identified as Gammaproteobacteria [16], whereas Clostridiales and* Methanobrevibacter* sp. dominated in the hindgut of* Melolontha melolontha *larvae [8]. Larvae of beetles of the families Cucujidae and Pyrochroidae are saproxylic and live under the bark [17]. Although the anatomy and physiology of many species of these families are well understood, little is known about the complex microbial communities associated with the gut systems and their importance for the beetles.

Larvae of longhorned beetles (family Cerambycidae) are xylophagous; they tunnel and feed in various tissues of healthy, dead, or decaying trees [3]. They can lead to a high mortality to both urban and forest trees, resulting in serious economic and environmental losses. Since the bark and wood are poor in amino-nitrogen but are rich in carbohydrates (cellulose and hemicelluloses) and lignin [18], many xylophagous insects carry a symbiotic complex that helps them to colonize such ecological niches. Previously, various representatives of the bacterial phyla Proteobacteria, Actinobacteria, Firmicutes, and Bacteroidetes have been identified in larvae and/or adults of several cerambycid beetles [3, 19]. More recently, high throughput sequencing analyses have been applied to identify bacteria associated with cerambycid beetles [20]. However, little is still known about the diversity, physiology, and ecology of bacteria and fungi living in the intestines of many other beetles which develop in the woody plants.

Deep interactions between beetles (especially beetles with limited diets) and microbes should be further investigated to understand the complex substrates (e.g., lignocellulosic biomass) metabolism. In addition to the fundamental interest of potential microbial roles in the ecology of the host organism, microbes in the insect intestine have proved to be important as a source of novel enzymes required in various industries and to be significant in new pest control strategies and degradation of lignocellulosic biomass [21]. In addition, the identification of microorganisms associated with larvae and/or adults of various beetles inhabiting various parts of the Russian Federation using high throughput sequencing approaches has been rarely performed.

The main aim of this research was to characterize the microbiota associated with the intestinal tract of several beetle larvae using deep sequencing of the bacterial/archaeal 16S rRNA genes and fungal internal transcribed spacer (ITS) region. Thus, larvae with various food preferences, including* Amphimallon solstitiale*,* Oryctes nasicornis*,* Cucujus cinnaberinus*,* Schizotus pectinicornis*,* Rhagium mordax*, and* Rhagium inquisitor*, were thoroughly studied to observe differences within prokaryotic and fungal communities inhabiting their intestinal tract.

## 2. Materials and Methods

### 2.1. Sample Collection and Preparation

Larvae of several beetles were collected from sites near city Kazan (Republic of Tatarstan, Russian Federation) in the summer of 2016. After their collection using tweezers, they were transported alive to the laboratory and maintained individually in sterile plastic containers together with soil, rotting manure, or wood pieces where they were discovered.* A. solstitiale* was collected from alfisol;* O. nasicornis* was obtained from rotting manure, whereas* C. cinnaberinus*,* S. pectinicornis*,* R. mordax*, and* R. inquisitor* were gently recovered from decaying trees. Initially second- or third-stage larvae of each species were grouped according to their morphological characteristics. Then, several representatives from each group were used for their further identification using molecular techniques (described below).

The microbiome associated with the intestinal tract was thoroughly investigated in this research. For each insect species, three individual larvae (three biological replicates) were investigated. The surface of individual larva was rinsed with 70% ethanol three times, washed twice in sterile phosphate-buffered saline to remove contamination and ethanol, and then dried for 1 min. The preparation of the intestinal tracts of larvae was performed on a sterilized glass slide with a pair of sterile tweezers and scalpel under sterile conditions. The experimental samples included the hindguts of root-feeding* A. solstitiale* larvae and rotting manure-feeding* O. nasicornis* larvae as well as the gut systems (midgut and hindgut) of other larvae. The content of gut systems of three selected larvae of each species was used for microbial genomic DNA extraction. The remaining larval cuticles were used for the host DNA extraction and taxonomic identification of insects.

### 2.2. Microbial DNA Extraction and PCR Amplification

Total DNA was extracted using a FastDNA SPIN kit for soil (MP Biomedicals) and a FastPrep-24 homogenizer (MP Biomedicals) according to the protocol provided by the manufacturer. The extracted DNA was quantified with a NanoDrop ND-2000 (Wilmington) and further used as a template for PCR using universal primers (constructed for Illumina sequencing) targeting bacterial and archaeal 16S rRNA gene and fungal ITS2 region. Bakt_341F (5′-CCT ACG GGN GGC WGC AG-3′) and Bakt_805R (5′-GAC TAC HVG GGT ATC TAA TCC-3′) primers were used to amplify V3 to V4 variable regions of the bacterial 16S rRNA gene [23]. Arch349F (5′-GYG CAS CAG KCG MGA AW-3′) and Arch806R (5′-GGA CTA CVS GGG TAT CTA AT-3′) primers were used to amplify V3 to V4 variable regions of the archaeal 16S rRNA gene [24]. ITS3_KYO2 (5′-GAT GAA GAA CGY AGY RAA-3′) and ITS4 (5′-TCC TCC GCT TAT TGA TAT GC-3′) primers were employed to amplify the fungal ITS2 region [25]. Each sample was amplified in triplicate and prepared for sequencing as described previously [26]. A negative control with sterile water performed during DNA extraction confirmed the absence of DNA contamination. The libraries containing 16S rRNA genes were sequenced using MiSeq v3 Reagent Kit (Illumina), while the libraries containing ITS region were sequenced using MiSeq v2 Reagent Kit (Illumina) on the MiSeq platform at Joint KFU-Riken Laboratory, Kazan Federal University (Kazan, Russia). Raw sequencing data are deposited in the NCBI's sequence read archive under accession numbers PRJNA393178 and PRJNA400790, and all data are available on request.

### 2.3. Larvae DNA Extraction, PCR Amplification, and Larvae Taxonomy

DNA from cuticles was extracted using a FastDNA SPIN kit (MP Biomedicals) and a FastPrep-24 homogenizer (MP Biomedicals) according to the protocol provided by the manufacturer. The extracted DNA was quantified with a NanoDrop ND-2000 (Wilmington). PCR amplicons obtained by using LCO-1480 (5′-GGT CAA CAA ATC ATA AAG ATA TTG G-3′) and HCO-2198 (5′-TAA ACT TCA GGG TGA CCA AAA AAT CA-3′) primers targeting cytochrome *c* oxidase (COI) subunit I gene [27] as well as CV7F (5′-CTT AAA GGA ATT GAC GGA GGG CAC CAC C-3′) and CV7R (5′-GAT TCC TTC AGT GTA GCG CGC GTG-3′) primers targeting the 18S rRNA gene [28] were purified with a QIAquick PCR Purification kit (Qiagen) and then sequenced on an ABI 3730 DNA Analyzer (Life Technologies). Sequences were uploaded to GenBank database which are available under accession numbers MF115589–MF115591, MF115593–MF115596, MF115598, MF776948, MF776956, MF776957, and MF776965.

### 2.4. Bioinformatic Analysis

Obtained Illumina data were analyzed using the bioinformatics pipeline QIIME, version 1.9.1 [29]. Illumina paired-end reads were initially assembled according to established protocols. Reads were then processed to eliminate low quality and chimeric sequences. The open reference-based operational taxonomic unit (OTU) picking strategy was applied to cluster reads into OTUs (at least five reads for an OTU; 97% identity threshold). For the taxonomic classification of the 16S rRNA gene sequences, the latest Greengenes database [30], and RDP Classifier [31] were used. For the taxonomic classification of the ITS sequences, UNITE database [32] was applied. In case of fungal data, all sequences not belonging to fungi were eliminated from further analysis. Low abundance sequences (relative abundance lower than 0.01%) were also further excluded. Alpha diversity was calculated at given numbers of reads (according to the sample containing the smallest set of reads) to equalize the sampling depth. OTU numbers, Shannon entropy, Simpson, Chao 1, and Fisher's alpha indices were estimated as the indicators for alpha diversity. NMDS analysis was performed on the sample-OTU matrix using the Bray–Curtis distances. The heat maps of the relative abundances of various taxa in the gut samples were generated using R package Vegan [33].

For larvae taxonomy, COI gene sequences were submitted to Barcode of Life Data System (BOLD) as a query for species identification, and 18S rRNA gene sequences were compared with the SILVA rRNA database. Species identification was also confirmed by BLAST searches in GenBank's database. In addition, all COI nucleotide sequences were translated to amino acid sequences to check for nuclear mitochondrial pseudogenes. Neighbor-joining trees of beetles were calculated with MEGA7 based on Kimura 2-parameter (K2P) distance [22].

## 3. Results

### 3.1. Identification of Species of Insects

The general identification of many species of insects can be complex and time-consuming, which is a limiting factor in the assessment of biodiversity. Moreover, the use of traditional morphological approaches for many taxa characterizations can be impossible when identifying larval stages of organisms due to the lack of diagnostic morphological characteristics and the presence of sibling species [28]. Therefore, in addition to the morphological methods in the present study, we analyzed the fragments of mitochondrial cytochrome *c* oxidase I (COI) gene for the accurate and reliable differentiation and identification of several Coleoptera species inhabiting the Republic of Tatarstan (Russia). Furthermore, we compared the COI gene datasets with the nuclear 18S rRNA gene (V7 region) datasets.

**Figure 1:**
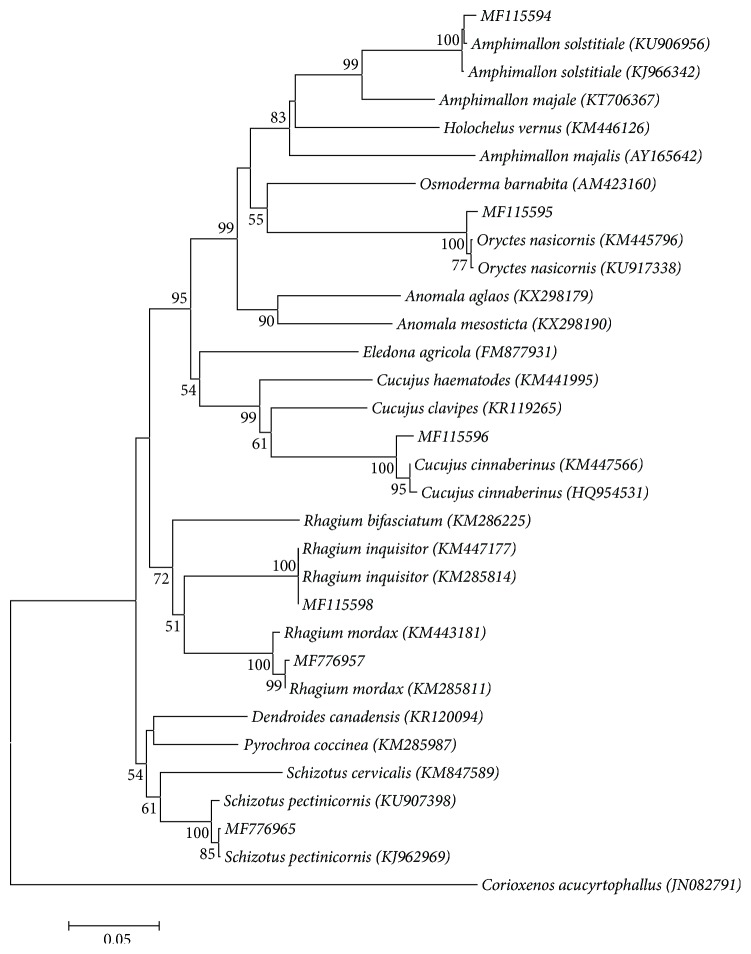
Neighbor-joining tree of COI sequence divergences (K2P) in the selected beetle species. The percentage of replicate trees in which the associated taxa clustered together in the bootstrap test (1000 replicates) are shown next to the branches. The analysis involved 32 nucleotide sequences. Evolutionary analyses were conducted in MEGA7 [22].* Corioxenos acucyrtophallus *(Strepsiptera) was used as an outgroup taxon.

We analyzed six species of five genera of beetles (larval stage). The mitochondrial COI region and nuclear region were successfully PCR amplified and sequenced in all cases, confirming the universality of these primers for the tested beetles. The neighbor-joining trees constructed from the obtained COI and 18S rRNA nucleotide sequences are illustrated in Figures 1 and S1 (Supplementary Information, SI), respectively. The applied mitochondrial COI gene allowed a better resolution, differentiation, and identification of larval stages of analyzed beetles. Based on the COI gene analysis we were able to confirm the identity of the following species:* Amphimallon solstitiale*,* Oryctes nasicornis*,* Cucujus cinnaberinus*,* Schizotus pectinicornis*,* Rhagium mordax*, and* Rhagium inquisitor*. Based on the data received during the application of 18S rRNA gene approach, it was possible to identify four species using the V7 marker. Therefore, 18S rRNA gene can be applied as a supplementary molecular marker to the COI barcode region for the tested beetles. Summarizing all results, our data demonstrate that COI gene is the most beneficial and effective molecular marker for identifying species of analyzed beetles.

### 3.2. Prokaryotic Communities Associated with Larval Guts

To investigate the microbial communities associated with some larvae with different food preferences, including* A. solstitiale*,* O. nasicornis*,* C. cinnaberinus*,* S. pectinicornis*,* R. mordax*, and* R. inquisitor*, their gut contents were recovered. Two* Rhagium* larvae have been previously investigated for their bacterial community composition [34]. For comparison, these data have been included in current analysis. More than 1.6 million high-quality-filtered sequences were obtained after processing of eighteen samples. In general, the amplicon sequencing sufficiently covered most of the bacterial phylotypes observed in all samples as it is indicated by the rarefaction curves (Figure S2, SI). Comparison of the alpha diversities between microbiome of different larvae was performed on equalized-sequence number (Table S1, SI). In general, the bacterial communities associated with larvae with different food preferences were diverse, and the bacterial community was characterized by a higher diversity in the samples retrieved from all* O. nasicornis* larvae.

**Figure 2:**
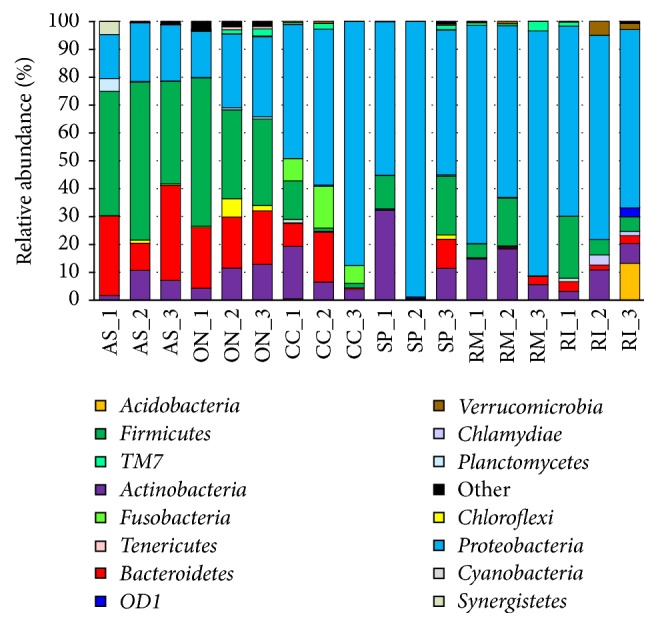
Relative abundance of bacterial phyla (based on 16S rRNA gene) within larval gut samples. Abbreviations of samples in figure in accordance with the scientific names of beetles (*A. solstitiale*: AS;* O. nasicornis*: ON;* C. cinnaberinus*: CC;* S. pectinicornis*: SP;* R. mordax*: RM;* R. inquisitor*: RI) and the order of the individual larva. Only phyla comprising at least 1% relative abundance in at least one sample are presented.

The relative abundance of specific bacterial groups was investigated at different taxonomic levels, that is, phylum, family, and genus. In total, 20 bacterial phyla were recovered from eighteen samples. Most of the identified OTUs belonged to the phyla Proteobacteria (16–98% of the total reads), Firmicutes (0.1–57%), Actinobacteria (0.5–32%), and Bacteroidetes (0.1–34%). The classification of the obtained bacterial sequences at the phylum level is demonstrated in Figure 2. Proteobacteria was found to be the most dominant phylum in the* R. mordax*,* R. inquisitor*,* S. pectinicornis*, and* C. cinnaberinus* larval guts. The Firmicutes phylotypes were found at higher levels in* A. solstitiale* and* O. nasicornis* larvae. Bacteroidetes was discovered as an additional substantial phylum in samples retrieved from* A. solstitiale* and* O. nasicornis* larvae, while the phylum Actinobacteria was also found at different levels in the gut samples retrieved from most investigated larvae. It should be also mentioned that phylotypes belonging to the phylum Fusobacteria were exclusively specific for* C. cinnaberinus* larvae. The other phyla representing more than 1% of the sequences at least in one of the samples were Acidobacteria, Chlamydiae, Chloroflexi, Cyanobacteria, Planctomycetes, Synergistetes, Tenericutes, and Verrucomicrobia as well as candidate divisions OD1 and TM7 (Figure 2).

On a family taxonomic scale, analysis of the most abundant phylotypes across samples from* A. solstitiale *hindgut revealed taxa related to the families Bacteroidaceae, Porphyromonadaceae (Bacteroidetes), Bacillaceae, Lachnospiraceae, Ruminococcaceae (Firmicutes), and Desulfovibrionaceae (Proteobacteria) but their abundance depended on the individual larva (Figure S3, SI). Microbial communities from the larval hindgut of* O. nasicornis* were mostly represented by unclassified Actinomycetales (Actinobacteria), Porphyromonadaceae, Rikenellaceae (Bacteroidetes), Turicibacteraceae, unclassified Clostridiales, Lachnospiraceae, Ruminococcaceae (Firmicutes), Desulfovibrionaceae, and Enterobacteriaceae (Proteobacteria). Representatives of the Leptotrichiaceae (Fusobacteria) and Enterobacteriaceae (Proteobacteria) were mostly detected in all* C. cinnaberinus *larvae. Bacterial communities in* S. pectinicornis* gut systems were also dominated by the common family Enterobacteriaceae (Proteobacteria) but were additionally represented by several other important groups and depended on the individual host organism. The bacterial community of three* R. mordax* individuals was also complex and comprised different families, including Microbacteriaceae, Cellulomonadaceae (Actinobacteria), Veillonellaceae (Firmicutes), Bradyrhizobiaceae, Rhizobiaceae, Comamonadaceae, Oxalobacteraceae, and Enterobacteriaceae (Proteobacteria), but their presence and abundance depended on the investigated larval gut (each larva was dominated by specific taxa). Sequence analysis additionally showed the complex community in the* R. inquisitor* larval gut as well. Thus, Bacillaceae (Firmicutes), Enterobacteriaceae, and Moraxellaceae (Proteobacteria) comprised the vast majority of the bacterial community in one* R. inquisitor* individual; Bradyrhizobiaceae, Comamonadaceae, and Oxalobacteraceae (Proteobacteria) were specific for the second individual, whereas Acidobacteriaceae (Acidobacteria), Acetobacteraceae, Burkholderiaceae, and Xanthomonadaceae (Proteobacteria) were abundant in the third* R. inquisitor* larva (Figure S3, SI).

**Figure 3:**
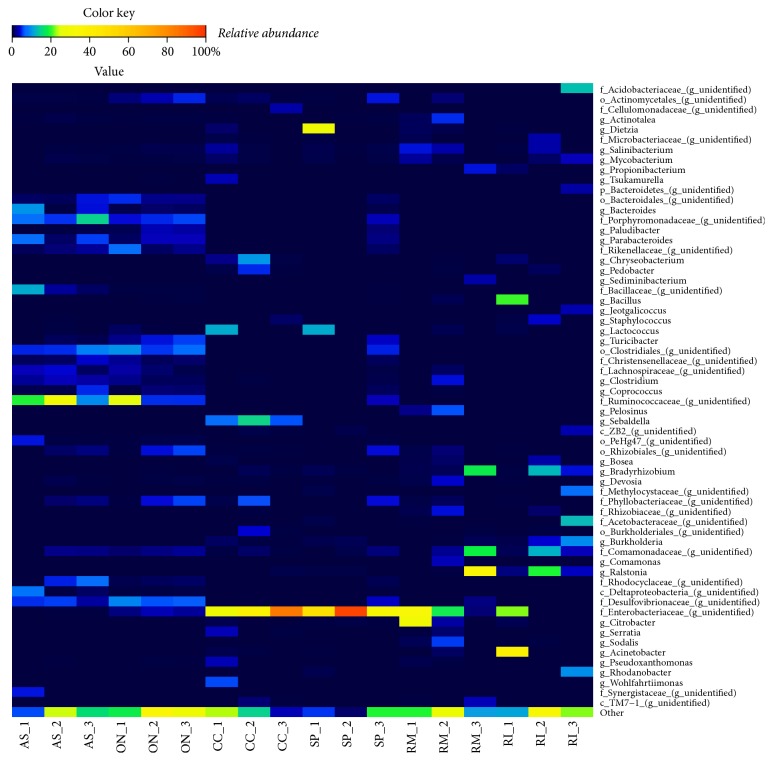
Heatmap illustrating the relative abundances of bacterial taxa among larval gut microbiota (genus level). Abbreviations of samples in figure in accordance with the scientific names of beetles (*A. solstitiale*: AS;* O. nasicornis*: ON;* C. cinnaberinus*: CC;* S. pectinicornis*: SP;* R. mordax*: RM;* R. inquisitor*: RI) and the order of the individual larva. Only taxa comprising at least 3% relative abundance in at least one sample are presented.

**Figure 4:**
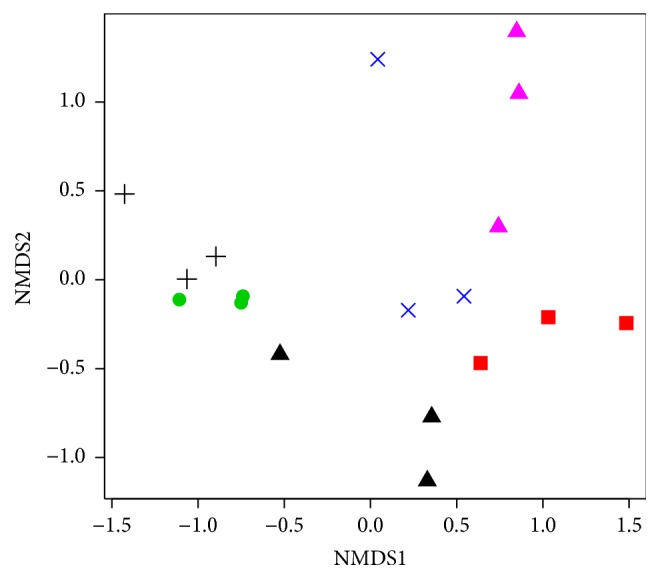
Nonmetric multidimensional scaling analysis of the Bray–Curtis dissimilarity index of the bacterial community OTUs (≥97% identity) based on Illumina sequencing of 16S rRNA genes. Symbols:* A. solstitiale*: black cross;* O. nasicornis*: green circle;* C. cinnaberinus*: red square;* S. pectinicornis*: black triangle;* R. mordax*: blue cross;* R. inquisitor*: magenta triangle (stress value: 0.15).

Figure 3 illustrates a heatmap of the relative abundance of the most abundant bacterial genera associated with the beetles' gut systems. We also compared the overall bacterial community structures using the NMDS analysis (Figure 4). Despite several dissimilarities between bacterial communities of one beetle species (as was reported above), this analysis mostly revealed the grouping of different replicates of one insect type together, though several samples of one beetle species were also close to some samples of the other beetle species. Furthermore, samples obtained from* R. mordax *and* R. inquisitor* were relatively scattered within the NMDS plot, indicating a more variability of their bacterial communities.

**Figure 5:**
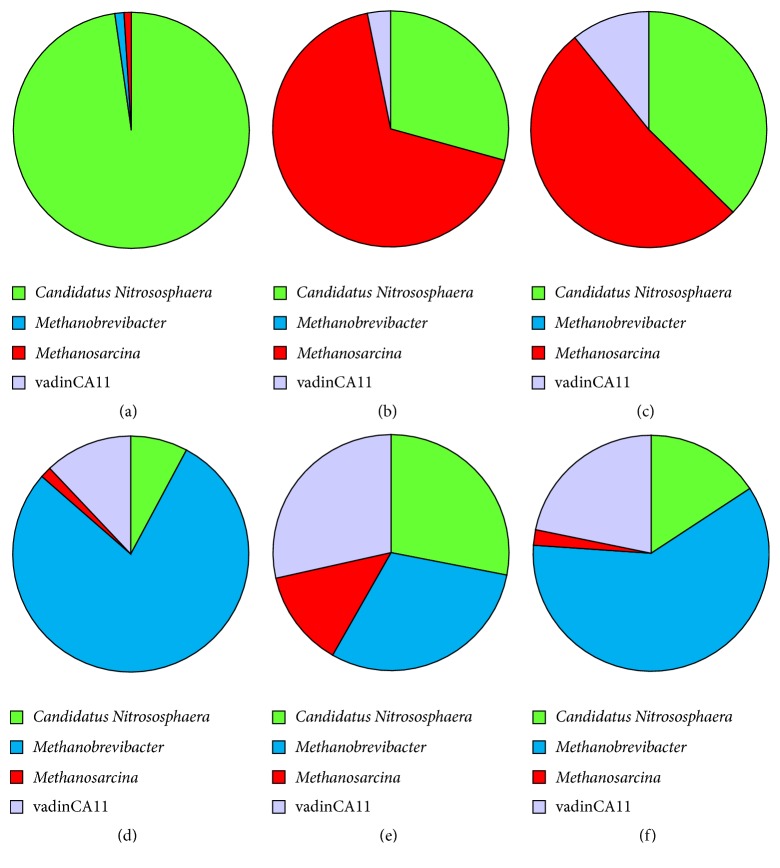
Relative abundance of archaeal genera (based on 16S rRNA gene) in* A. solstitiale* ((a) AS_1; (b) AS_2; (c) AS_3) and* O. nasicornis* ((d) ON_1; (e) ON_2; (f) ON_3) larval hindgut samples.

With the applied methods we were able to amplify archaeal 16S rRNA gene fragments from hindgut samples of* O. nasicornis* and at lower levels from hindgut samples of* A. solstitiale*, whereas in all other cases no archaeal 16S rRNA genes could be amplified or they were amplified at insufficient level to perform Illumina sequencing. Taxonomic analyses of the most abundant OTUs across* O. nasicornis* and* A. solstitiale* samples revealed taxa related to the phyla* Euryarchaeota* and* Crenarchaeota*. More than 30,000 high-quality-filtered sequences were obtained after processing of six samples of* O. nasicornis* and* A. solstitiale* larvae, and the analysis also showed the variation of archaea in their gut systems. In the hindgut of various* A. solstitiale* larvae higher levels of* Candidatus* Nitrososphaera and* Methanosarcina* and lower levels of candidate genus vadinCA11 were observed (Figure 5). The archaeal communities in the hindguts of* O. nasicornis* larvae were dominated by methanogenic archaea belonging to the genus* Methanobrevibacter*. Furthermore, notable levels of* Candidatus *Nitrososphaera, candidate genus vadinCA11, and* Methanosarcina *were detected (Figure 5).

### 3.3. Fungal Communities Associated with Larval Guts

To characterize the fungal community associated with larvae of several beetles, the fungal ITS2 region was amplified with the primers that preferentially target the fungal internal transcribed spacer region [25]. More than 1.1 million high-quality fungal sequences were generated after processing of seventeen samples (one sample was excluded from analysis due to the low sequencing depth). In general, the ITS sequencing sufficiently covered most of the fungal taxa found in seventeen samples as it is indicated by the rarefaction curves (Figure S4). Comparison of the alpha diversities between the fungal communities of different larvae was elucidated on equalized-sequence number.  Table S2 (SI) shows alpha diversity indices, which were calculated to investigate the biodiversity of the fungal community in the larval gut samples. In general, the fungal communities associated with various beetle larvae were considerably less diverse than bacterial communities, and fungal community was found to be more diverse in the gut samples retrieved from* O. nasicornis *and less diverse in* R. inquisitor* samples.

**Figure 6:**
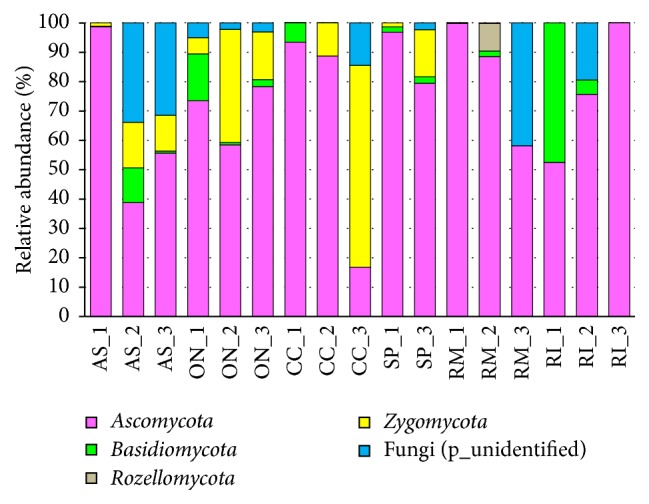
Relative abundance of fungal phyla (based on ITS region) within larval gut samples. Abbreviations of samples in figure in accordance with the scientific names of beetles (*A. solstitiale*: AS;* O. nasicornis*: ON;* C. cinnaberinus*: CC;* S. pectinicornis*: SP;* R. mordax*: RM;* R. inquisitor*: RI) and the order of the individual larva (SP_2 sample was excluded from analysis due to the low sequencing depth).

The relative abundance of specific fungal groups was elucidated at different taxonomic levels, such as phylum, family, and genus. The classification analysis of the obtained fungal sequences at the phylum level is demonstrated in Figure 6. Most of the obtained fungal OTUs were related to the phyla Ascomycota (17–99% of the total reads), Basidiomycota (0–47%), Zygomycota (0–68%), and Rozellomycota (0–9%) as well as to unidentified fungi (0–42%). In total, 4 fungal phyla were obtained from seventeen gut samples. Ascomycota was found to be the most prevalent phylum in most of the* A. solstitiale*,* O. nasicornis*,* C. cinnaberinus*,* S. pectinicornis*,* R. mordax*, and* R. inquisitor* individuals with the exception of one* C. cinnaberinus* larva.

**Figure 7:**
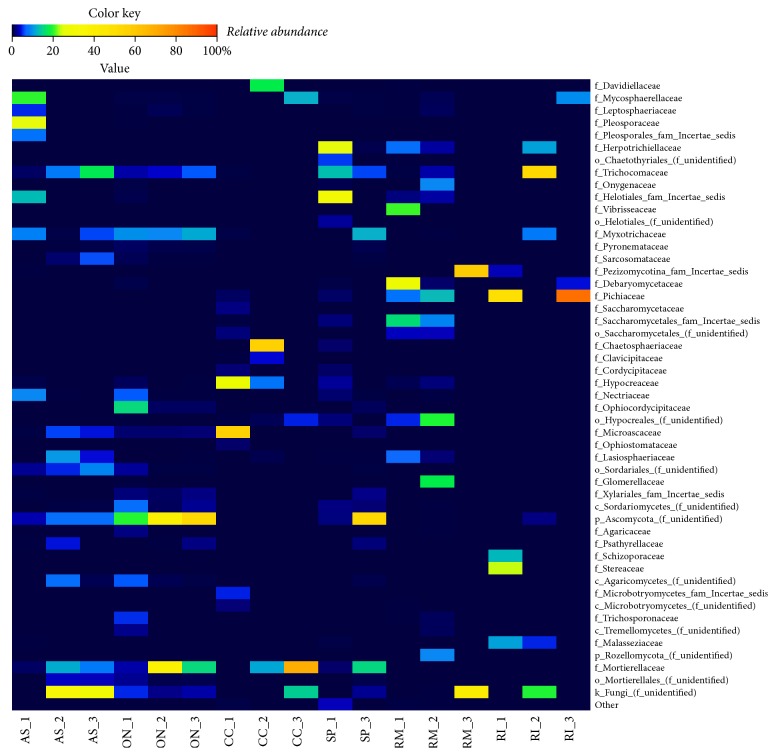
Heatmap illustrating the relative abundance of fungal taxa among larval gut mycobiome (family level). Abbreviations of samples in figure in accordance with the scientific names of beetles (*A. solstitiale*: AS;* O. nasicornis*: ON;* C. cinnaberinus*: CC;* S. pectinicornis*: SP;* R. mordax*: RM;* R. inquisitor*: RI) and the order of the individual larva (SP_2 sample was excluded from analysis due to the low sequencing depth). Only taxa comprising at least 2% relative abundance in at least one sample are presented.

On a family taxonomic scale, the abundant fungal OTUs in the gut samples retrieved from all insects species were also depended on the beetle species and individual host organism. Thus, in samples retrieved from one* A. solstitiale* larval gut we primarily identified OTUs related to the families Mycosphaerellaceae, Pleosporaceae, Helotiales–Family Incertae sedis, and Nectriaceae (Ascomycota), while other two larvae were dominated by unidentified fungi as well as by different other families (Figure 7). The fungal consortia in the larval gut of* O. nasicornis* were dominated by unknown Ascomycota and the common families Myxotrichaceae, Trichocomaceae (Ascomycota), and Mortierellaceae (Zygomycota). Members of the Microascaceae and Hypocreaceae (Ascomycota) were specific for one* C. cinnaberinus* larva, Chaetosphaeriaceae, Davidiellaceae (Ascomycota), and Mortierellaceae (Zygomycota) for the second individual, whereas Mortierellaceae (Zygomycota) dominated in the third larva (up to 69%). In case of* S. pectinicornis* larvae, Helotiales–Family Incertae sedis and Herpotrichiellaceae (Ascomycota) were abundant in one representative, while unclassified Ascomycota and Mortierellaceae (Zygomycota) were observed at high levels in the other larva. The fungal communities in the guts of* Rhagium* were also highly diverse. Debaryomycetaceae and Vibrisseaceae (Ascomycota) were specific for one* R. mordax* larva; unknown Hypocreales, Glomerellaceae, and Onygenaceae (Ascomycota) were mostly observed in the second larva, while Pezizomycotina–Family Incertae sedis and unidentified fungi were found at high levels in the third individual. In case of* R. inquisitor*, two individuals harbored a high proportion of Pichiaceae (Ascomycota), whereas the fungal communities in another animal were dominated by Trichocomaceae (Ascomycota) (Figure 7).

**Figure 8:**
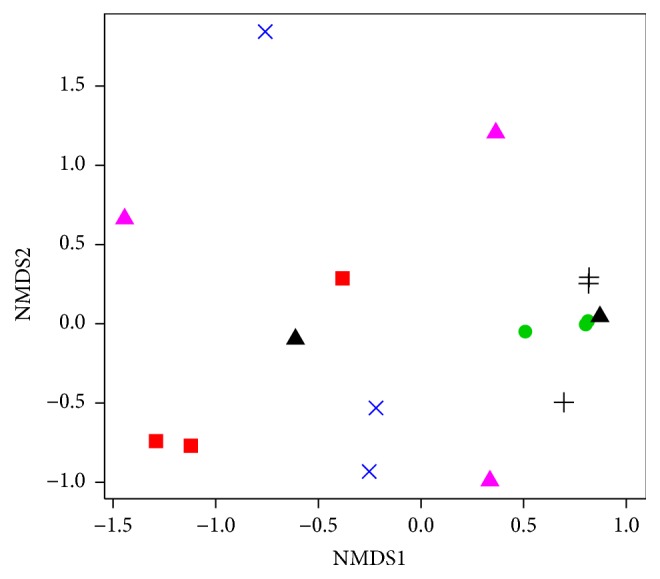
Nonmetric multidimensional scaling analysis of the Bray–Curtis dissimilarity index of the fungal community OTUs (≥97% identity) based on Illumina sequencing of ITS region. Symbols:* A. solstitiale*: black cross;* O. nasicornis*: green circle;* C. cinnaberinus*: red square;* S. pectinicornis*: black triangle;* R. mordax*: blue cross;* R. inquisitor*: magenta triangle (stress value: 0.16). SP_2 sample was excluded from analysis due to the low sequencing depth.

Figure S5 (SI) shows the most abundant fungal genera detected in individual gut mycobiome. We also compared the overall fungal community structure using NMDS analysis (Figure 8). Unlike bacterial communities, this analysis revealed a high variability of fungal communities within individual larvae with the exception of samples retrieved from* A. solstitiale* and* O. nasicornis*.

## 4. Discussion

The study described herein shows the prokaryotic and fungal communities associated with the intestinal systems of several beetle larvae. Thus, larvae with different food preferences, including* A. solstitiale*,* O. nasicornis*,* C. cinnaberinus*,* S. pectinicornis*,* R. mordax*, and* R. inquisitor*, were accurately studied to see differences between microbial consortia inhabiting their intestinal tract, which may help in the utilization of their nutritional resources. Enzymes necessary for the transformation of several organic compounds can be produced either by the host arthropod itself, or by microbes from the intaken feed or by symbiotic microorganisms in their intestines [21]. Our data also confirm the high potential of COI barcodes as well as 18S rRNA gene (V7 region) for identification of species of these Coleoptera species.

The applied analysis revealed that the composition of intestinal bacteria and fungi in the studied Coleoptera larvae has distinct microbial OTUs differences. In this study, we found an association of analyzed beetle species mainly with four bacterial phyla, Proteobacteria, Firmicutes, Actinobacteria, and Bacteroidetes, and with three fungal phyla, Ascomycota, Zygomycota, and Basidiomycota, but their proportion varied depending on the beetle host, individual organism, and surrounding environment. The dominance of such bacterial [2, 3, 19, 20] and fungal phyla [3, 6] has also been reported in the previous works devoted to the identification of gut microbiota in different other insect species. Regarding the archaeal communities, with the applied techniques we could distinguish archaeal OTUs of the phyla Euryarchaeota and Crenarchaeota in hindgut samples of* O. nasicornis* and* A. solstitiale*.

### 4.1. Prokaryotic Communities of Several Coleoptera

Our characterization of the hindgut microbiota of phytophagous* A. solstitiale* larvae (Scarabaeidae) by 16S rRNA gene sequencing revealed the genera* Bacteroides* and* Parabacteroides* as well as some Porphyromonadaceae, Bacillaceae, Ruminococcaceae, and Desulfovibrionaceae clades at notable levels. It should be mentioned that several representatives of the Porphyromonadaceae, Ruminococcaceae, and Desulfovibrionaceae as well as unclassified Clostridiales were also abundant in saprophagous/xylophagous* O. nasicornis* larvae (Scarabaeidae). Interestingly, most of these phylotypes could not be detected or were discovered at very low levels in all other investigated Coleoptera species in this research. Carbohydrate fermentation by* Bacteroides* bacteria leads to the production of a pool of volatile fatty acids (VFA) which can be potentially used by hosts as energy sources [21, 35]. Species of the Porphyromonadaceae can synthesize various VFA from carbohydrates or proteins [36]. Members of the family Ruminococcaceae hydrolyze a pool of polysaccharides by various mechanisms, for example, through the production of a cellulosomal enzyme complex as well as cellulose-binding proteins [37]. They can ferment hexoses and pentoses which are the hydrolysis products derived from cellulose and hemicelluloses, respectively, and the functional role of Ruminococcaceae representatives in both Scarabaeidae larval guts seems to be mostly the digestion of cellulolytic material. Such clades of microbes (e.g., some lineages of Porphyromonadaceae and Ruminococcaceae) are also found within other insects but are unknown or rarely reported in the free-living condition [21]. Several other cellulolytic bacteria as well as cellulases have also been identified in the larval guts of* Holotrichia parallela* [5, 38] and* Oryctes rhinoceros* (Scarabaeidae) [39]. Moreover, bacterial-mediated cellulose destruction has also been implicated in the gut of European rhinoceros beetle larvae [15]. The presence of Desulfovibrionaceae phylotypes in the hindgut content of* A. solstitiale* and* O. nasicornis* suggests an important physiological role of sulfate-reducing bacteria in hindgut metabolism.* Desulfovibrio* species have also been previously discovered in the* M. melolontha* (Scarabaeidae) intestine [8].

Regarding archaeal communities, we were able to distinguish archaeal OTUs in hindguts samples only from scarab beetles. Thus, the archaeal communities in the hindgut of* O. nasicornis* larvae were dominated by methanogens belonging to the strict hydrogenotrophic genus* Methanobrevibacter*, producing methane from either H_2_/CO_2_ or formate [40]. In addition, notable levels of ammonia-oxidizing* Candidatus* Nitrososphaera, candidate genus vadinCA11 as well as acetoclastic, hydrogenotrophic, and methylotrophic* Methanosarcina* spp. [11] were discovered. Ammonia-oxidizing archaea oxidize ammonia to nitrite with its further oxidation to nitrate by nitrite-oxidizing bacteria [41]. In* A. solstitiale* larvae ammonia-oxidizing* Candidatus *Nitrososphaera and mixotrophic* Methanosarcina* as well as lower levels of candidate genus vadinCA11 were observed. Archaea mostly are not associated with insects, although several members were reported to be found in insects and were prevalent in the hindguts of other larvae of scarab beetles, cockroaches, and termites [8, 9].

Saproxylic larvae (*Cucujus *and* Schizotus* species) live under the bark and depend on the presence of decaying wood, other saproxylic organisms, or wood fungi [17, 42]. The two saproxylic species shared many of the bacterial taxa with the Enterobacteriaceae being predominant family, but the overall bacterial community profile differed due to the differences in their habitats and diet regime. Thus, unclassified Enterobacteriaceae as well as several other important taxa were detected at high levels in all* C. cinnaberinus* and* S. pectinicornis* larval guts. Moreover, the genus* Sebaldella* was exclusively specific for* C. cinnaberinus* larvae and observed at substantial levels. Enterobacteriales species have already been found in other beetles' guts, where they were involved in metabolism of polysaccharides as well as nitrogen-fixing processes [43]. Obligately anaerobic* Sebaldella termitidis* is the only species in the genus* Sebaldella* (the order Fusobacteriales) isolated previously from intestinal content of termites. This microorganism may play a role in providing nitrogen to the termite host and can produce acetic and lactic acids from different sugars [44]. Instead of anaerobic clostridial phylotypes, mainly specific for both Scarabaeidae larvae studied in this work, another group of hydrolytic bacteria (Actinomycetales) was observed in most our Cucujidae and Tenebrionoidea larvae. Actinomycetes can be used in the biological pretreatment of the lignocellulosic substrate [45].

The bacterial community structure of xylophagous longhorn beetle larvae (between individuals) was very complex compared with the previously described Coleoptera species. Two larvae of* R. mordax* comprised high levels of various Enterobacteriaceae members, while the abundant bacteria in the third* R. mordax* larva were closely related to the genera* Ralstonia* and* Bradyrhizobium*. Sequence analysis additionally showed the complex community in the* R. inquisitor* larval gut as well.* Bacillus*,* Acinetobacter*, and unclassified Enterobacteriaceae comprised the vast majority of the bacterial community in one* R. inquisitor* individual;* Ralstonia* and* Bradyrhizobium *were specific for the second individual, whereas unknown Acidobacteriaceae, unknown Acetobacteraceae,* Burkholderia*, and* Rhodanobacter* were abundant in the third* R. inquisitor* larva. Bacteria of the Enterobacteriaceae [43] and* Bacillus* [46] could be potentially involved in different polysaccharides (also cellulose) metabolism as well as nitrogen-fixing processes. Different members of the Enterobacteriales were also abundant in* R. inquisitor* larvae identified with the 16S rRNA cloning/sequencing techniques [3]. Species of the genus* Bradyrhizobium* [47] and several species of the genera* Ralstonia* and* Burkholderia* [48] isolated from different ecological niches are also capable of nitrogen fixation. Bacteria of the Acidobacteriaceae are all acidophilic and able to degrade a wide array of carbon compounds as well as plant and microbial polysaccharides, including cellulose [49].* Rhodanobacter* species can utilize various carbon sources, including cellobiose [50]. It is interesting to note that clostridia were rarely identified in the gut of xylophagous* R. inquisitor* larvae.

Bacterial metabolism of the substrates can lead to the formation of a pool of various compounds which can potentially be used by insects. Thus, it is possible that several bacteria identified in this research work were involved in insects' substantial nutritional roles. However, the presence of these microorganisms in larval guts could be additionally associated with the composition of the feeding material (can be derived from the feed of the larvae) and the individual living place of insects, as the substrates on which the larvae feed can be the major determinants for the gut microbial content. Although environmentally obtained microbes may on many occasions be transient associates (from feeding material), several specific and functionally significant interactions can be developed de novo in every host generation by the acquiring of stable microorganisms from the environment where they live.

### 4.2. Fungal Communities of Several Coleoptera

In addition to bacterial gut community,* A. solstitiale* larvae (feeding on the roots of herbaceous plants) were mostly associated with Ascomycota, including representatives of the genera* Mycosphaerella* and* Chalastospora* (one larva), unknown fungi and unknown Ascomycota (other two larvae), as well with many other important genera (but their presence and abundance depended on the individual organism). It should be also mentioned that the genera* Penicillium*,* Pseudogymnoascus*, unclassified Sordariales, unclassified Ascomycota, and the genus* Mortierella* were specific for all three* Amphimallon *larvae (but were detected at lower levels).* Penicillium* species have an enzymatic machinery to degrade lignocellulosic material, such as beta-exoglucanase, beta-endoglucanase, beta-glucosidase, and other enzymes [51, 52]. Several* Pseudogymnoascus* species are reported as cellulolytic, saprotrophic, and psychrophilic [53]. However, it was also reported that* Pseudogymnoascus destructans* as a representative of the genus Pseudogymnoascus could infect hibernating bats [54]. The fungal communities from the gut of* O. nasicornis* larvae were predominated by the common genus* Pseudogymnoascus* and unknown Ascomycota, whereas within Zygomycota, members of the family Mortierellaceae were detected at notable levels. Many of identified fungi have been also previously detected in the gut contents of various beetle species [55–57]. Several fungi detected in the gut content of both scarab beetles could also be plant associated fungi. All these fungi and several other could enter the gut of* A. solstitiale* and* O. nasicornis* larvae with the feeding material, and some of them might perform mutualistic and/or pathogenic interactions.

Saproxylic* Cucujus *and* Schizotus* larvae depend on dead/dying trees, the presence of other saproxylic organisms, or wood fungi [17, 42]. Members of the genera* Graphium* and* Trichoderma* were mainly detected in one* C. cinnaberinus* larva;* Chloridium*,* Cladosporium*,* Trichoderma*, and* Mortierella* were observed in the second individual, whereas* Mortierella* dominated in the third* Cucujus *larva. Fungi in the genus* Graphium* are found in different environments, including soil and woody substrates, and some of them can decompose lignin [58]. Moreover, the presence of* Graphium euwallaceae* was observed in larvae and adult beetles of* Euwallacea fornicatus* (Coleoptera, Scolytinae) as well in the galleries of several tree species [59] (the similar living environment for* C. cinnaberinus*).* Trichoderma* species are common plant saprophytes and many of them are strongly cellulolytic; they are often observed on cellulosic materials such as decaying wood and various woody products and can produce different organic acids [60]. In several wood-feeding Coleoptera larvae found in tropical forests of Costa Rica,* Trichoderma* was the most abundant genus as well [61], comprising a number of glycoside hydrolases, peroxidases, and laccases which are involved in the degradation of lignocellulose materials [62]. Several species of this genus are also known as biological control agents against various fungal diseases of plants [63].* Chloridium paucisporum* as a representative of the genus* Chloridium* can form ectendomycorrhizae in pine [64], while species of the genus* Mortierella* live as saprotrophs in the soil, on decaying organic material, and possess the cellulolytic activity [65]. The genus* Mortierella* was also detected in most samples (except for the male adults) of the* Dendroctonus armandi* (Scolytinae) gut-associated fungal community [56]. The genera* Leptodontidium*,* Capronia*, and* Penicillium *were mostly observed in one* S. pectinicornis* larva, whereas unknown Ascomycota and unknown Mortierellaceae were specific for another larva.* Leptodontidium* species have been previously cultivated on a wide range of carbohydrates, including cell-wall-related compounds [66]. Most species of the genus* Capronia* occur on rotting wood or bark and the decaying parts of herbaceous plants [67], and several strains are able to degrade hemicellulose [68]. Most of these fungi and several other could enter the gut of analyzed* Cucujus *and* Schizotus* larvae with the feeding material and serve as the additional nutrient source and/or they might perform various interactions.

The fungal communities in the guts of* Rhagium* varied between individuals. Thus, fungal OTUs detected in one* R. mordax* were closely related to the genera* Scheffersomyces*,* Phialocephala*,* Cyberlindnera*, and* Capronia*. Unknown Hypocreales and the genera* Colletotrichum*,* Ogataea*, and* Auxarthron* were mostly observed in second larva, while the genus* Chalara* and unidentified fungi were found at high levels in third individual. In case of* R. inquisitor*, two individuals harbored a high proportion of the genus* Hyphopichia*, whereas the fungal communities in another animal were dominated by* Penicillium* and* Talaromyces* genera.* Ogataea polymorpha* (as a representative of the genus* Ogataea*) is one of the most important industrially applied yeasts, which can ferment xylose and has been studied as a potential producer of ethanol from biomass with high lignocellulosic content [69, 70].* Hyphopichia* species can be detected in different environments; they are also associated with beetles or beetle larval substrates and can ferment different types of sugars [71]. In case of xylophagous* R. inquisitor* larvae, only several ascomycetous yeasts strains could be isolated from their gut content in previous work performed by Grünwald et al. [3]. Several carrion beetles have also been reported to harbor a diversity of ascomycetous yeasts closely related to* Yarrowia lipolytica* [6], which is represented as major biotechnological interest for bioremediation of contaminated environments [72, 73]. Several other filamentous fungi and yeasts observed in the guts content of* Rhagium* were also detected in several other environments (including soil, woody substrates, and beetles' gut systems) and might be involved in transformation of various types of substrates (carbohydrates, proteins, and lipids).

In addition to the complex composition of bacterial communities, each species of Coleoptera investigated in the research described herein harbored various fungi belonging mainly to the phyla Ascomycota, Zygomycota, and Basidiomycota. Some specific and functionally significant interactions can be developed de novo in every host generation by the acquiring of stable associates from the environment where they live. The identification of many inhabitants with their potential to perform complex substrates conversion suggests that particular fungal groups might play substantial roles in providing nutritional needs for the hosts. Also, fungi themselves could serve as the additional nutrient source for some Coleoptera species. However, the presence of these fungi in larval guts could be additionally associated with the composition of the feeding material and the individual feeding place of insects, as the substrates on which the larvae feed can be major determinants for the gut mycobiome content.

## 5. Conclusions

In conclusion, the present study characterizes the microbial communities associated with the gut systems of several larvae (Coleoptera). In this research, we revealed an association of all beetle species mainly with four bacterial phyla, Proteobacteria, Firmicutes, Actinobacteria, and Bacteroidetes, as well as with three fungal phyla, Ascomycota, Zygomycota, and Basidiomycota, but their proportion varied depending on the investigated beetle host, individual organism, and surrounding environment. In addition, with the applied methods we were able to distinguish archaea belonging to the phyla Euryarchaeota and Crenarchaeota in the hindgut content of* O. nasicornis* and* A. solstitiale*. The metabolic potential of the identified microorganisms suggests a possible role in the exploitation of various resources, providing nutritional needs for the host organism. The beetle-associated microbes can also be a valuable source of novel metabolic capacities for their application in various biotechnologies. Future directions will be aimed at isolating the detected bacteria and fungi and analyzing the whole metagenome in order to investigate the functions in which they are involved.

## Supplementary Materials

Supplementary Materials Figure S1: neighbor-joining tree of the V7 region (18S rRNA gene) datasets for several beetles (based on Kimura 2-parameter method). The percentage of replicate trees in which the associated taxa clustered together in the bootstrap test (1000 replicates) are shown next to the branches. The analysis involved 27 nucleotide sequences. Evolutionary analyses were conducted in MEGA7.* Corioxenos acucyrtophallus* (Strepsiptera) was used as an outgroup taxon. Figure S2: rarefaction curves of the sequencing data of the bacterial 16S rRNA gene amplicons. As the rarefaction curves demonstrate, the depth of the conducted amplicon sequencing covered sufficiently most of the bacterial phylotypes present in the eighteen analyzed larval gut samples. Table S1: summary of observed bacterial OTUs, Shannon entropy, Simpson, Chao 1, Fisher's alpha, and phylogenetic diversity values. Figure S3: relative abundance of bacterial 16S rRNA genes among various larval gut samples (family level). Abbreviations of samples in figure in accordance with the scientific names of beetles (*A. solstitiale*: AS;* O. nasicornis*: ON;* C. cinnaberinus*: CC;* S. pectinicornis*: SP;* R. mordax*: RM;* R. inquisitor*: RI) and the order of the individual larva. Only taxa comprising at least 2% relative abundance in at least one sample are presented. Figure S4: rarefaction curves of the sequencing data of the fungal ITS amplicons. As the rarefaction curves demonstrate, the depth of the conducted amplicon sequencing covered sufficiently most of the fungal phylotypes present in the seventeen analyzed larval gut samples. One sample (SP_2) was excluded from analysis due to the low sequencing depth. Table S2: summary of observed fungal OTUs, Shannon entropy, Simpson, Chao 1, and Fisher's alpha indices. Figure S5: relative abundance of fungal ITS sequences among various larval gut samples (genus level). Abbreviations of samples in figure in accordance with the scientific names of beetles (*A. solstitiale*: AS;* O. nasicornis*: ON;* C. cinnaberinus*: CC;* S. pectinicornis*: SP;* R. mordax*: RM;* R. inquisitor*: RI) and the order of the individual larva (SP_2 sample was excluded from analysis due to the low sequencing depth). Only taxa comprising at least 2% relative abundance in at least one sample are presented.
