# Supplementary material for: Fungal, Bacterial, and Archaeal Diversity in the Digestive Tract of Several Beetle Larvae (Coleoptera)
Source: Biomed Res Int. 2018 Apr 16;2018:6765438. doi: 10.1155/2018/6765438 (PMC5926521; doi:10.1155/2018/6765438)
Supplement: Supplementary Materials — Figure S1: neighbor-joining tree of the V7 region (18S rRNA gene) datasets for several beetles (based on Kimura 2-parameter method). The percentage of replicate trees in which the associated taxa clustered together in the bootstrap test (1000 replicates) are shown next to the branches. The analysis involved 27 nucleotide sequences. Evolutionary analyses were conducted in MEGA7. Corioxenos acucyrtophallus (Strepsiptera) was used as an outgroup taxon. Figure S2: rarefaction curves of the sequencing data of the bacterial 16S rRNA gene amplicons. As the rarefaction curves demonstrate, the depth of the conducted amplicon sequencing covered sufficiently most of the bacterial phylotypes present in the eighteen analyzed larval gut samples. Table S1: summary of observed bacterial OTUs, Shannon entropy, Simpson, Chao 1, Fisher's alpha, and phylogenetic diversity values. Figure S3: relative abundance of bacterial 16S rRNA genes among various larval gut samples (family level). Abbreviations of samples in figure in accordance with the scientific names of beetles (A. solstitiale: AS; O. nasicornis: ON; C. cinnaberinus: CC; S. pectinicornis: SP; R. mordax: RM; R. inquisitor: RI) and the order of the individual larva. Only taxa comprising at least 2% relative abundance in at least one sample are presented. Figure S4: rarefaction curves of the sequencing data of the fungal ITS amplicons. As the rarefaction curves demonstrate, the depth of the conducted amplicon sequencing covered sufficiently most of the fungal phylotypes present in the seventeen analyzed larval gut samples. One sample (SP_2) was excluded from analysis due to the low sequencing depth. Table S2: summary of observed fungal OTUs, Shannon entropy, Simpson, Chao 1, and Fisher's alpha indices. Figure S5: relative abundance of fungal ITS sequences among various larval gut samples (genus level). Abbreviations of samples in figure in accordance with the scientific names of beetles (A. solstitiale: AS; O. nasicornis: ON; C. c [file 6765438.f1.pdf]

Supplementary information to the article entitled:

## Fungal, Bacterial and Archaeal Diversity in the Digestive Tract of Several Beetle Larvae (Coleoptera)

Authors are:

Elvira E. Ziganshina, Waleed S. Mohammed, Elena I. Shagimardanova, Petr Y. Vankov, Natalia E. Gogoleva and Ayrat M. Ziganshin

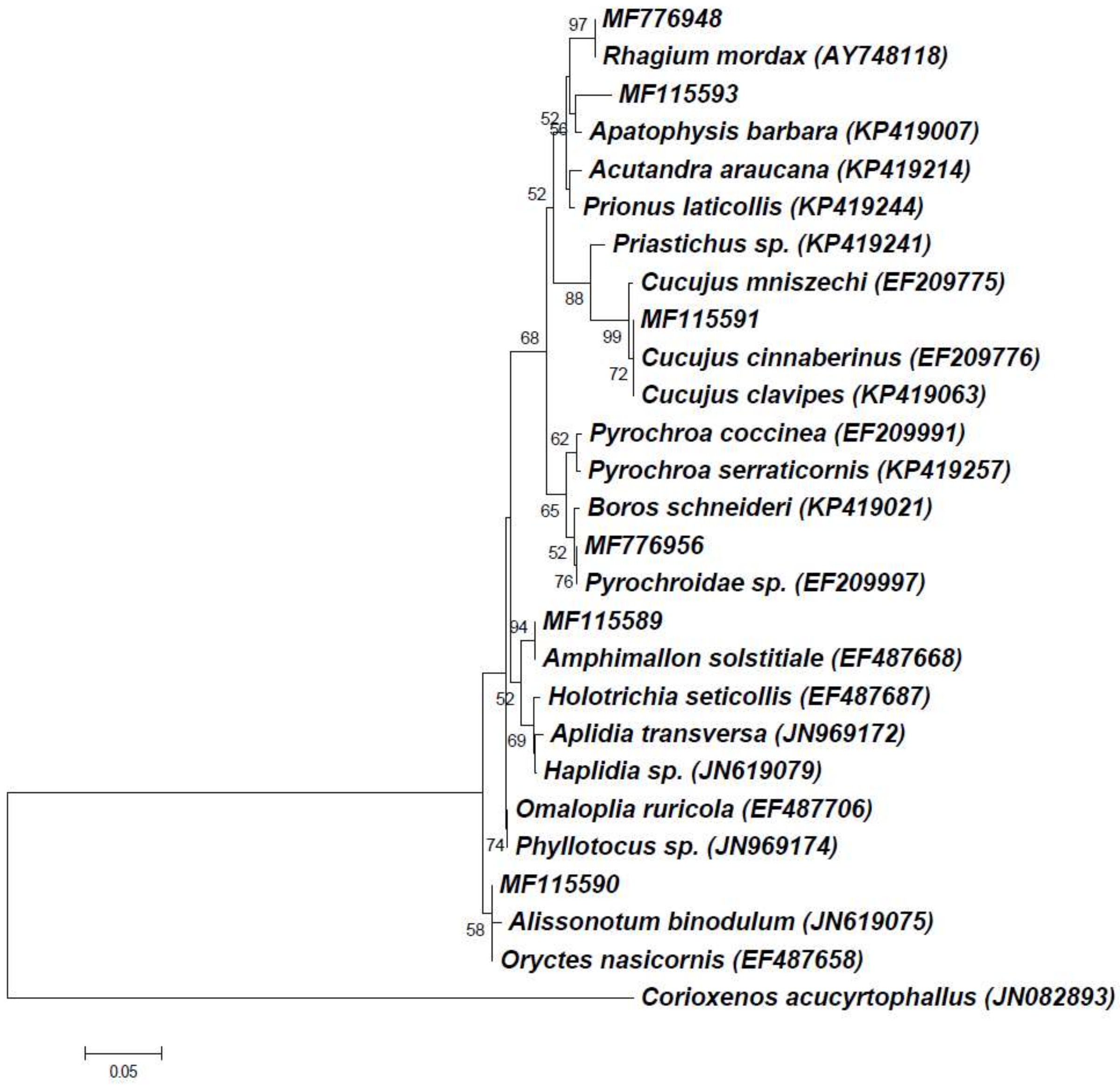


**Figure S1.** Neighbor-joining tree of the V7 region (18S rRNA gene) datasets for several beetles (based on Kimura 2-parameter method). The percentage of replicate trees in which the associated taxa clustered together in the bootstrap test (1000 replicates) are shown next to the branches. The analysis involved 27 nucleotide sequences. Evolutionary analyses were conducted in MEGA7. *Corioxenos acucyrtophallus* (Strepsiptera) was used as an outgroup taxon.

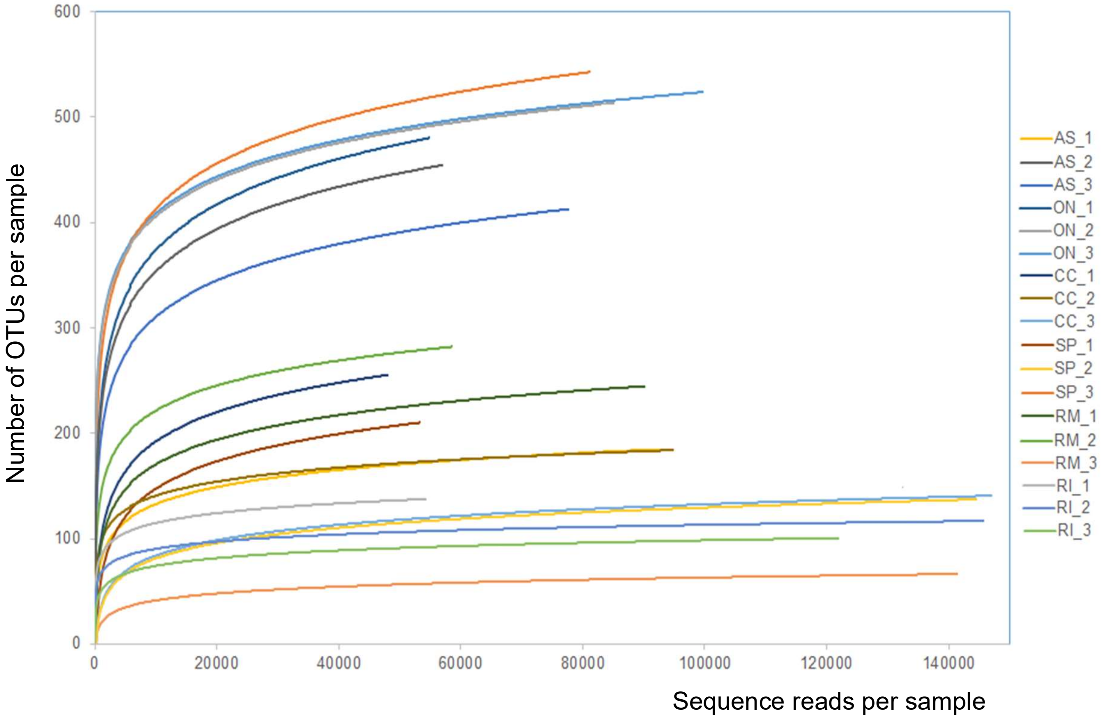


**Figure S2.** Rarefaction curves of the sequencing data of the bacterial 16S rRNA gene amplicons. As the rarefaction curves demonstrate, the depth of the conducted amplicon sequencing covered sufficiently most of the bacterial phylotypes present in the eighteen analyzed larval gut samples.

**Table S1.** Summary of observed bacterial OTUs, Shannon entropy, Simpson, Chao 1, Fisher's alpha and phylogenetic diversity values.

| Sample ID | OTUs | Shannon entropy | Simpson | Chao 1 | Fisher's alpha | PD Whole Tree |
|---|---|---|---|---|---|---|
| AS_1 | 168 | 5.29 | 0.95 | 213.09 | 22.11 | 15.97 |
| AS_2 | 433 | 6.95 | 0.98 | 478.12 | 66.67 | 33.41 |
| AS_3 | 388 | 6.65 | 0.98 | 450.64 | 58.58 | 29.91 |
| ON_1 | 465 | 7.05 | 0.99 | 510.00 | 72.54 | 37.30 |
| ON_2 | 478 | 7.36 | 0.99 | 505.35 | 74.95 | 37.92 |
| ON_3 | 483 | 7.18 | 0.98 | 509.71 | 75.88 | 37.65 |
| CC_1 | 256 | 5.02 | 0.91 | 278.94 | 36.01 | 20.01 |
| CC_2 | 173 | 4.43 | 0.91 | 186.00 | 22.87 | 12.45 |
| CC_3 | 118 | 2.21 | 0.65 | 148.00 | 14.75 | 12.16 |
| SP_1 | 204 | 3.04 | 0.79 | 243.05 | 27.67 | 13.83 |
| SP_2 | 115 | 1.47 | 0.43 | 140.67 | 14.32 | 10.21 |
| SP_3 | 502 | 6.17 | 0.93 | 543.25 | 79.44 | 39.13 |
| RM_1 | 225 | 4.20 | 0.86 | 257.22 | 30.99 | 15.98 |
| RM_2 | 270 | 6.15 | 0.97 | 297.00 | 38.31 | 16.38 |
| RM_3 | 57 | 3.76 | 0.87 | 102.33 | 6.46 | 7.81 |
| RI_1 | 131 | 4.12 | 0.89 | 141.00 | 16.62 | 9.90 |
| RI_2 | 102 | 5.32 | 0.95 | 115.75 | 12.49 | 9.19 |
| RI_3 | 82 | 5.12 | 0.96 | 87.60 | 9.74 | 7.74 |

Alpha diversity was calculated at given number of reads 44,029.

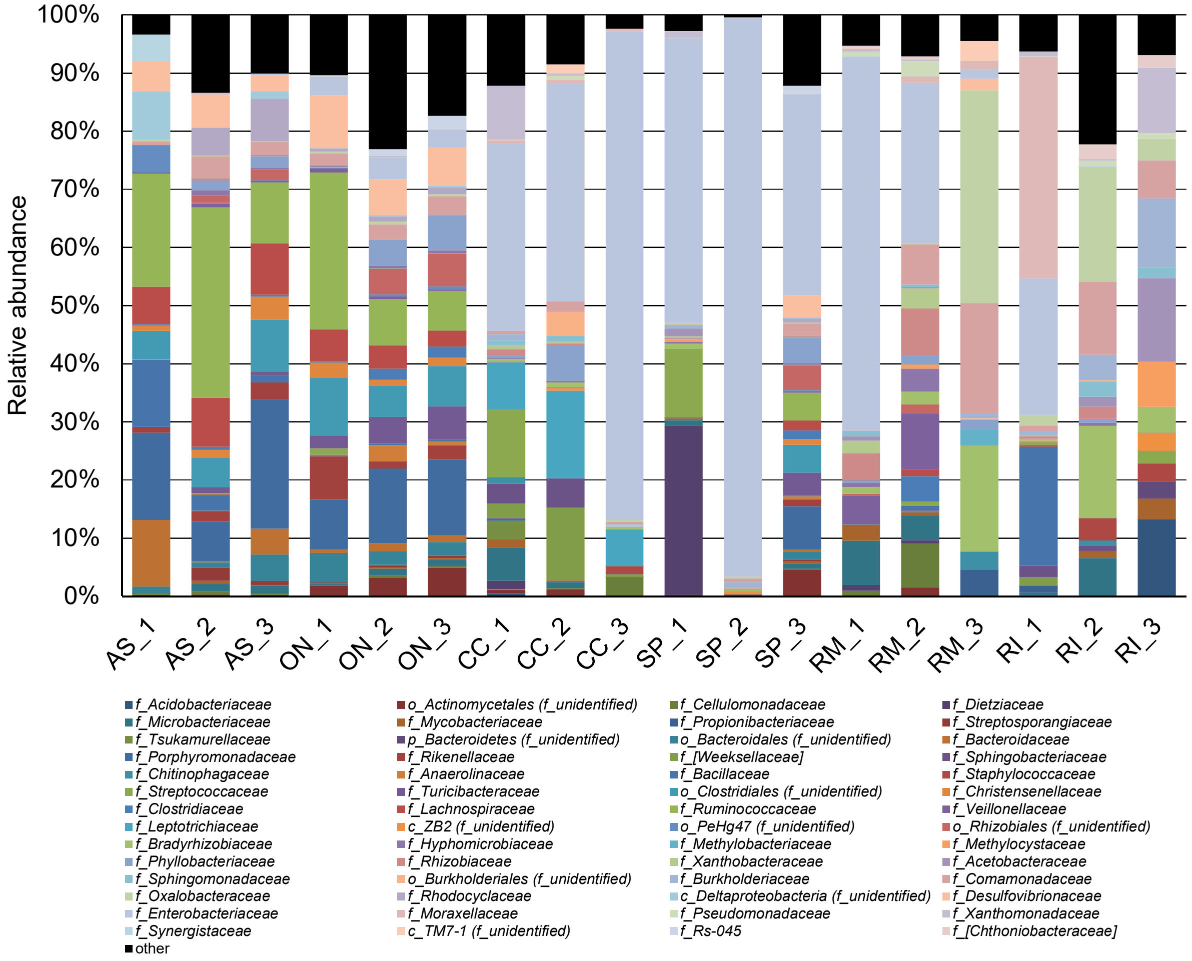


**Figure S3.** Relative abundance of bacterial 16S rRNA genes among various larval gut samples (family level). Abbreviations of samples in figure in accordance with the scientific names of beetles (*A. solstitiale* – AS; *O. nasicornis* – ON; *C. cinnaberinus* – CC; *S. pectinicornis* – SP; *R. mordax* – RM; *R. inquisitor* – RI) and the order of the individual larva. Only taxa comprising at least 2% relative abundance in at least one sample are presented.

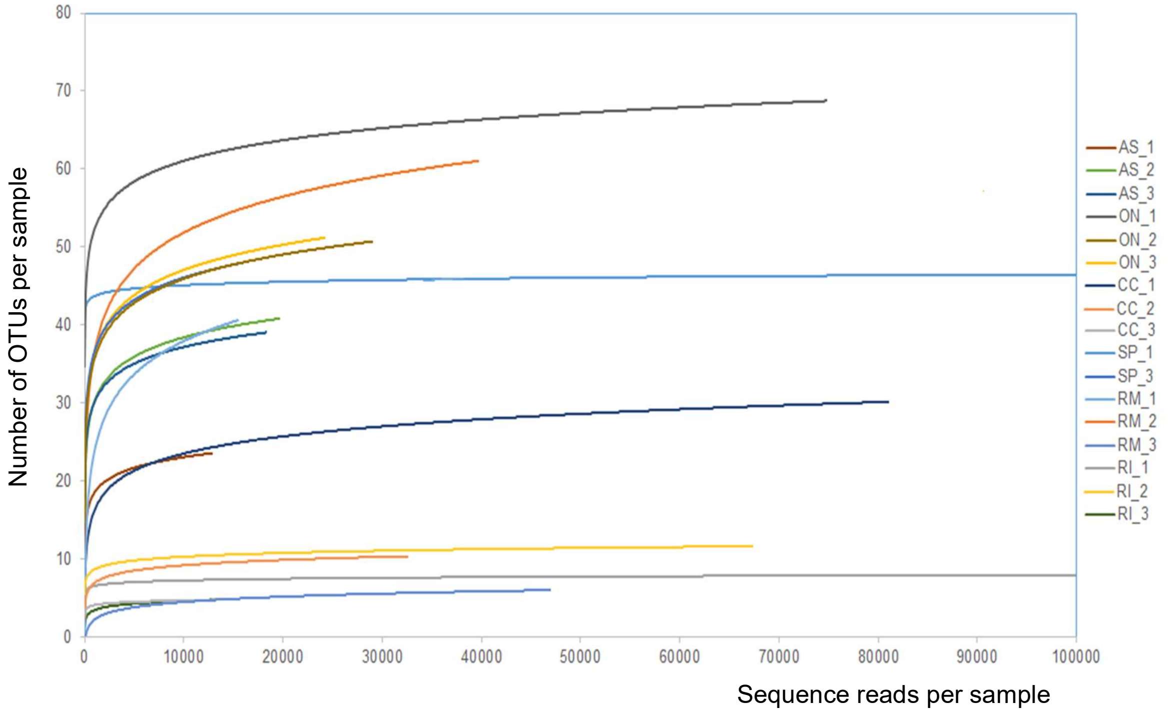


**Figure S4.** Rarefaction curves of the sequencing data of the fungal ITS amplicons. As the rarefaction curves demonstrate, the depth of the conducted amplicon sequencing covered sufficiently most of the fungal phylotypes present in the seventeen analyzed larval gut samples. One sample (SP_2) was excluded from analysis due to the low sequencing depth.

**Table S2.** Summary of observed fungal OTUs, Shannon entropy, Simpson, Chao 1 and Fisher's alpha indices.

| Sample ID | OTUs | Shannon entropy | Simpson | Chao 1 | Fisher's alpha |
|---|---|---|---|---|---|
| AS_1 | 23 | 3.07 | 0.85 | 24.00 | 2.74 |
| AS_2 | 38 | 3.86 | 0.91 | 41.00 | 4.85 |
| AS_3 | 39 | 3.88 | 0.91 | 42.00 | 5.00 |
| ON_1 | 65 | 4.58 | 0.94 | 67.50 | 9.01 |
| ON_2 | 46 | 3.29 | 0.81 | 46.50 | 6.04 |
| ON_3 | 47 | 3.64 | 0.87 | 48.00 | 6.19 |
| CC_1 | 24 | 1.94 | 0.61 | 25.00 | 2.87 |
| CC_2 | 8 | 1.86 | 0.63 | 8.00 | 0.83 |
| CC_3 | 5 | 1.35 | 0.49 | 5.00 | 0.49 |
| SP_1 | 46 | 3.75 | 0.85 | 46.00 | 6.04 |
| SP_3 | 45 | 3.60 | 0.86 | 45.00 | 5.89 |
| RM_1 | 40 | 3.11 | 0.84 | 43.00 | 5.15 |
| RM_2 | 55 | 3.68 | 0.89 | 62.00 | 7.43 |
| RM_3 | 4 | 1.38 | 0.56 | 4.00 | 0.39 |
| RI_1 | 7 | 1.98 | 0.68 | 7.00 | 0.72 |
| RI_2 | 10 | 2.68 | 0.82 | 10.00 | 1.07 |
| RI_3 | 5 | 0.72 | 0.25 | 5.00 | 0.49 |

Alpha diversity was calculated at given number of reads 12,211. One sample (SP_2) was excluded from analysis due to the low sequencing depth.

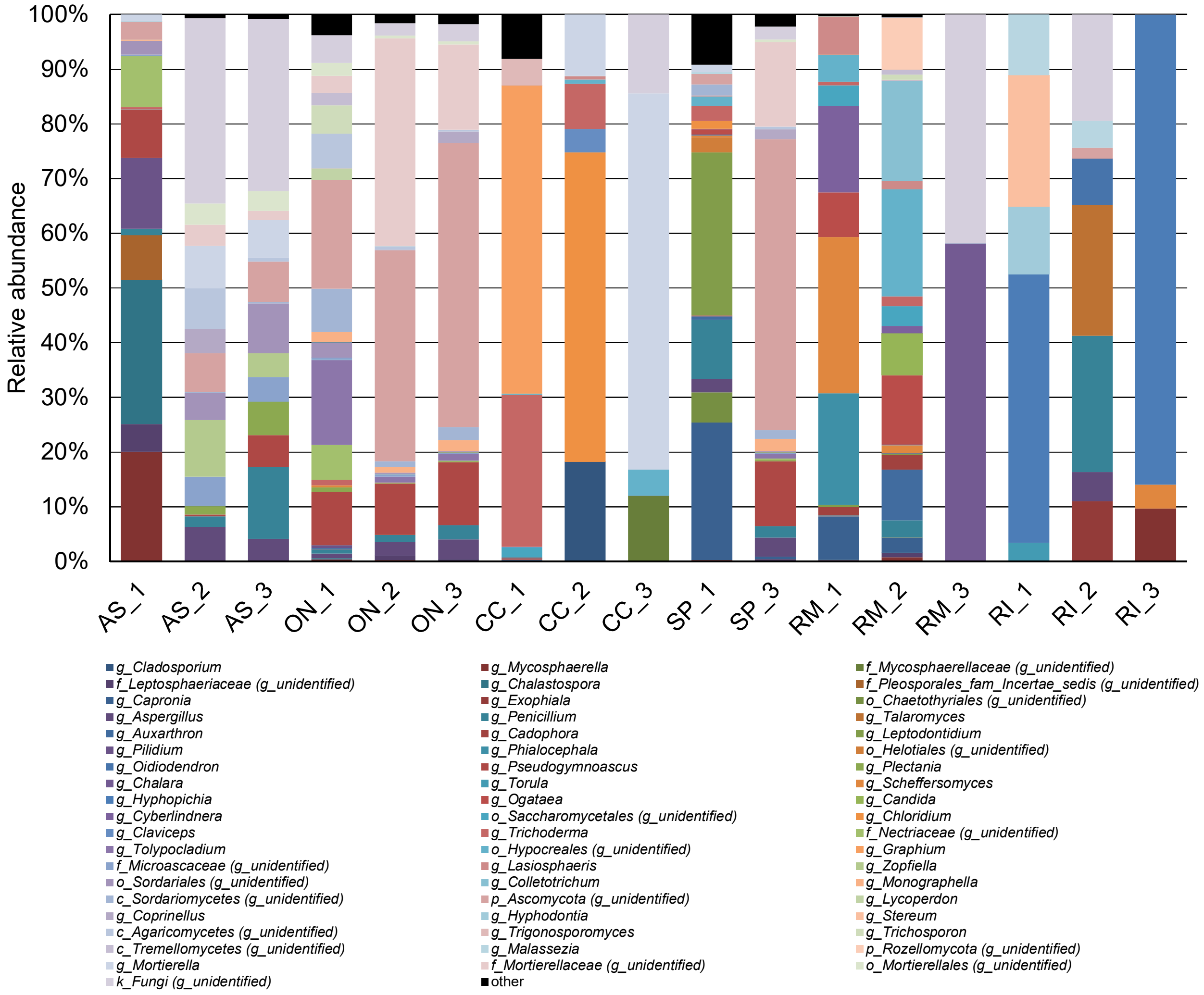


**Figure S5.** Relative abundance of fungal ITS sequences among various larval gut samples (genus level). Abbreviations of samples in figure in accordance with the scientific names of beetles (*A. solstitiale* – AS; *O. nasicornis* – ON; *C. cinnaberinus* – CC; *S. pectinicornis* – SP; *R. mordax* – RM; *R. inquisitor* – RI) and the order of the individual larva (SP_2 sample was excluded from analysis due to the low sequencing depth). Only taxa comprising at least 2% relative abundance in at least one sample are presented.
